# The Effects of WW2/WW3 Domains of Smurf2 Molecule on CD4^+^CD25^+^/CD4^+^ Proportion in Spleen of 4T1 Tumor Bearing BALB/c Mice

**DOI:** 10.29252/ibj.24.4.214

**Published:** 2020-02-10

**Authors:** Hani Mosayebzadeh Roshan, Seyed-Hosein Abtahi-Eivary, Hassan Shojaee-Mend, Alireza Mohammadzadeh, Zahra Bahari Sani

**Affiliations:** 1 Department of Basic Sciences, Faculty of Medicine, Gonabad University of Medical Sciences, Gonabad, Iran;; 2 Department of Medical Laboratory Sciences, Faculty of Paramedical Sciences, Gonabad University of Medical Sciences, Gonabad, Iran

**Keywords:** Arginase, Transforming growth factor-β, WW domains

## Abstract

**Background:**

TGF-β has long been considered as the main inducer of Tregs in tumor microenvironment and is the reason for the aberrant number of Tregs in tumor-bearing individuals. Recently, it has been suggested that the enzyme arginase I is able to mediate the induction of Tregs in a TGF-β-independent fashion. The recombinant WW2/WW3 domains from Smurf2 molecule was demonstrated to increase TGF-β signaling while reducing arginase I gene expression. In this study, we aimed to examine the effects of this recombinant protein on CD4^+^CD25^+^/CD4^+^ proportion in the spleen of 4T1 mammary carcinoma-bearing BALB/c mice.

**Methods:**

Flow cytometry was used to evaluate CD4^+^CD25^+^ spleen cell populations of the tumor-bearing mice that received WW2/WW3 protein treatment and those of the control group.

**Results:**

The results indicated a significant rise in CD4^+^CD25^+^/CD4^+^ ratio, along with an average increase in tumor mass of the subjects that underwent protein treatment.

**Conclusion:**

It can be inferred that the heightened CD4^+^CD25^+^/CD4^+^ proportion in the spleen of protein-treated tumor-bearing mice can be the result of the increased TGF-β signaling despite the reduced arginase I expression.

## INTRODUCTION

TGF-β is produced by a variety of cell types present in tumor microenvironment, leading to the induction of CD4^+^CD25^+^ regulatory T cells, which, in turn, produce more TGF-β and contribute to immunosuppression^[^^1^^-^^3^^]^. One of the mechanisms of immunosuppression is the recruitment of Gr-1^+^ CD11b^+^ MDSC into tumor microenvironment^[^^4^^]^. A major mechanism whereby these cells exert their immunosuppressive effects is the expression of the enzyme arginase I^[^^5^^,^^6^^]^. Arginase I acts to suppress immunity by depleting the tumor microenvironment from arginine, and this arginine-deprived condition rapidly compromises T cell function and proliferation at the tumor site due to a blockade in CD3ζ chain expression^[^^7^^,^^8^^]^. A previous investigation has demonstrated that the depletion of arginase I-producing MDSC in 4T1 mammary adenocarcinoma-bearing mice with antibody treatment reduces tumor size and metastasis to a certain extent but still not comparable with the group treated by anti-TGF-β antibody, in which a significant reduction of tumor size and metastasis was observed^[^^9^^]^. It has also been suggested in the same study that TGF-β neutralization could decrease MDSC numbers in tumors, along with decreasing arginase I gene expression in these cell types; all these changes coincided with a shift of tumor-associated myeloid cells̕ (MDSC) phenotype from inhibitory to activatory. These data show that although TGF-β works synergistically with arginase I, it stands out to be superior to arginase I with regard to its immunosuppressive effects.

The smurf2 molecule, a cytoplasmic E3 ubiquitin ligase, contains three separate WW domains designated as WW1, WW2, and WW3, each comprising of 33 amino acids in length. The name WW refers to the highly conserved tryptophan residues within the sequences. Among these domains, WW2 and WW3 are critical for the interaction with the PY motif of Smad7, a negative regulator of TGF-β signaling^[^^10^^]^. In our study, we used the recombinant WW2/WW3 domain as a smad7 binder. It has been shown that the recombinant WW2/WW3, carrying a linker between domains, is capable of binding to the Smad7 PY motif both *in vitro* and *in vivo*^[^^10^^,^^11^^]^ and altering the TGF-β intracellular signaling when introduced into the cytoplasm^[^^11^^]^. 

 TGF-β induces the formation of Treg cells by either the conversion of CD4^+^CD25^-^ into CD4^+^CD25^+^ T cells or proliferating the CD4^+^CD25^+^ Treg cell populations at the periphery^[^^1^^,^^12^^,^^13^^]^. TGF-β-independent induction of CD4^+^CD25^+^ Treg cells by arginase I as well as the induction of CD4^+^CD25^+^ Treg cells by arginase I-producing MDSC have already been described^[^^14^^,^^15^^]^. Therefore, arginase I has been proposed as an inducer of CD4^+^CD25^+^ Treg cells. In our previous study, we found that the recombinant WW2/WW3 protein could significantly reduce arginase I gene expression, while increasing TGF-β signaling^[^^11^^]^. In the current study, we aimed to examine the impacts of WW2/WW3 recombinant protein on spleen CD4^+^CD25^+^/CD4^+^ proportion in 4T1 tumor-bearing mice. In other words, the influence of the diminished arginase I expression and heightened TGF-β signaling, two opposing phenomena with regard to CD4^+^CD25^+^ T cells induction, on CD4^+^CD25^+^/CD4^+^ proportion was evaluated by applying the WW2/WW3 recombinant protein.

4T1 cell line has been used as a murine model of human breast cancer^[^^16^^]^. This tumor is proved to foster the secretion of TGF-β and expression of arginase I in the tumor microenvironment^[^^17^^,^^18^^]^. The general outline of this study was to evaluate the *in vivo* effects of the recombinant WW2/WW3 domains fused to TAT peptide (a cell-penetrating peptide that renders the whole protein capable of penetrating into cells in a receptor-independent fashion) on the ratio of spleen CD4^+^CD25^+^/CD4^+^ cell and tumor growth in 4T1 tumor-bearing mice

## MATERIALS AND METHODS

**Animals**

Female BALB/c mice were purchased from Pasteur Institute of Iran (Tehran). All animals were housed and maintained in a pathogen-free environment and were seven weeks old at the initiation of the animal studies. 

**Cell line and cell culture**

4T1, an adherent metastatic mammary carcinoma cell line derived from BALB/c mice, was obtained from Pasteur Institute of Iran, Tehran. The cell line was cultured in RPMI 1640 (Gibco, USA) medium containing 2 mM of glutamine under humidified 5% CO_2_ atmosphere at 37 ºC and supplemented with 10% heat-inactivated FBS (Gibco), as well as 100 U/mL of penicillin and 100 µg/mL of streptomycin (both from Sigma, St. Louis, MO, USA). Cells were allowed to reach 80% confluency in T-25 culture flasks and then trypsinized, washed and resuspended in 1× PBS buffer before being injected into the mice.

**WW2/WW3-TAT recombinant protein**

WW2/WW3-TAT recombinant protein is composed of the domains WW2 and WW3, along with the linker sequence between them from Smurf2 molecule fused to TAT peptide. This recombinant protein was expressed using pET21b+ plasmid in BL21 codon+ bacteria as described earlier^[^^11^^]^.

**Animal model and treatments protocol**

Two equal groups of five mice were designated as control and test groups. All the mice received 2.5 × 10^5^ 4T1 tumor cells in their hind flank on day zero. *In vivo* treatment was begun on day 10 by the intraperitoneal administration of 1 mg/kg recombinant WW2/WW3-TAT protein at each injection in the test group, and 1× PBS in the control group. The treatment was continued for two weeks, and each mouse received a total dose of seven injections. After the injection schedule was accomplished, all the mice were euthanized, their spleens were removed for harvesting splenocytes, and the tumors were removed and weighed.

**Antibodies and sample preparation for flow cytometry**

FITC-conjugated anti-mouse CD4 and PE-conjugated anti-mouse CD25 and corresponding isotype-matched controls were purchased from R&D systems (Minneapolis, Minnesota, USA). Single-cell suspensions of splenocytes were prepared by pressing the spleens between two slides and passing the suspension through a fine-bore needle. Erythrocytes were lysed by suspending the cells in ammonium-chloride-potassium lysing buffer on ice for 10 minutes. After washing with cold PBS, the harvested splenocytes were made into suspensions in a blocking buffer (2% FBS in PBS) and kept for 15 minutes at room temperature. For staining, antibodies were used in a concentration of 10 µl/10^6^ cells in FACS buffer (0.5% BSA in PBS) at room temperature for 30 minutes, according to the instructions provided by the manufacturer. After washing, the samples were fixed with the fixation buffer (1% paraformaldehyde in PBS) and analyzed on a FACSCalibur™ flow cytometer (BD Biosciences, USA). 

**Statistical analysis**

Data were analyzed using Mann–Whitney U Test. All the analyses were carried out using GraphPad Prism version 6.07 (San Diego, CA). *p* values less than 0.05 were considered statistically significant.

**Ethical statement**

The above-mentioned sampling protocols were approved by the Research Ethics Committee of Gonabad University of Medical Sciences, Gonabad, Iran (ethical code: GMU.REC.1393.151). 

## RESULTS

**CD4** ^+^ **CD25** ^+^ **and CD4** ^+^ **CD25** ^-^ **populations among spleen lymphocytes**

**Fig. 1:**
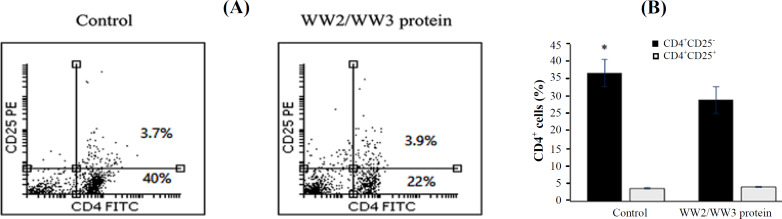
Decrease of CD4^+^CD25^-^T cells in tumor-bearing mice treated with the protein. (A) A representative flow cytometric dot plot analysis. Lymphocytes from treatment and control groups were gated by plotting the cells on forward vs. side scatter, followed by analysis for the expression of CD4 vs. CD25. (B) There was a significant reduction in the percentage of CD4^+^CD25^-^ cells in the group treated with WW2/WW3 protein compared with the control group (^*^p ˂ 0.05), but no significant change was observed in CD4^+^CD25^+^ cell populations between the two groups

In order to determine the impact of WW2/WW3 recombinant protein on splenocytes populations, 

CD4^+^CD25^-^ lymphocytes, CD4^+^CD25^+^ subset, and the CD4^+^CD25^+^/total CD4^+^ ratio were determined on each subject using anti-mouse CD4 and CD25 antibodies. To assess the percentage of CD4^+^CD25^-^ and CD4^+^CD25^+^ T cell population, lymphocytes were gated by plotting forward vs. side scatter, followed by analyzing CD4 vs. CD25 expression. The results indicated no change in CD4^+^CD25^+^ cells in the test group treated with the protein compared with the control group (Fig. 1). However, the reduction in CD4^+^CD25^-^ populations in the group treated with the protein was significantly notable (*p ˂* 0.05; Fig. 1). These data also suggested the proportionate increase of CD4^+^CD25^+^ T cells to CD4^+^CD25^-^ cells in the treatment group as compared to the control group. 

**CD4** ^+^ **CD25** ^+^ **/total CD4** ^+^ **ratio**

**Fig. 2:**
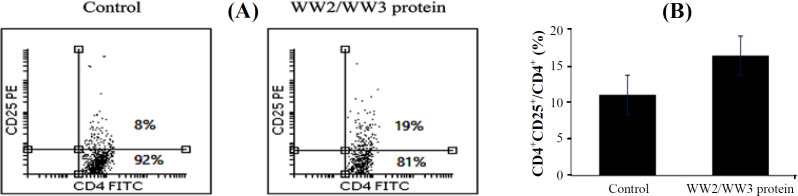
Increase of CD4^+^CD25^+^/CD4^+^ ratio in the protein treatment category of tumor-bearing mice. (A) A representative flow cytometric dot plot analysis comparing two subjects from treatment and control groups. CD4-expressing lymphocytes were gated by plotting the cells on side scatter vs. CD4, followed by the analysis for CD4 vs. CD25 expression. (B) There was a significant increase in CD4^+^CD25^+^/CD4^+^ ratio in the protein treatment group (*p* ˂ 0.05)

The major index in evaluating spleen CD4^+^CD25^+^ cells is to determine CD4^+^CD25^+^/total CD4^+^ ratio. To do so, total splenocytes were plotted against side scatter vs. CD4, which was followed by gating on CD4-expressing T cells. The gated cell population was analyzed for CD4 vs. CD25 expression. The results showed an increase in the CD4^+^CD25^+^/total CD4^+^ ratios in the treatment group (*p* ˂ 0.05; Fig. 2). Increase in CD4^+^CD25^+^/total CD4^+^ ratio among tumor-bearing individuals was associated with tumor advancement and suggestive of a more suppressive functionality in the immune systems of this group of tumor-bearing mice treated with the protein.

**Tumor growth evaluation**

**Fig. 3:**
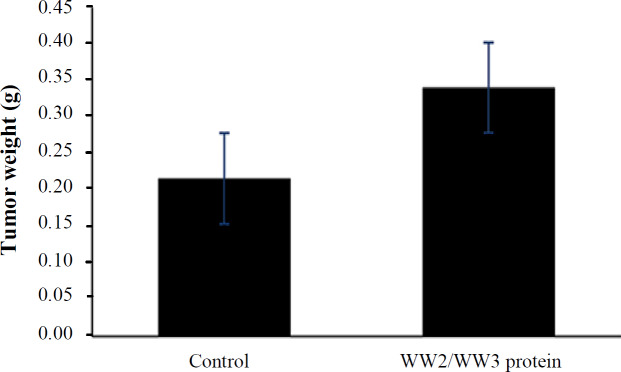
*In vivo* treatment of 4T1 tumor-bearing mice with the recombinant protein, resulting in 61% higher tumor growth compared with the untreated controls (*p* = 0.08)

After treatment, the mice were euthanized, the tumors were excised, and their weights were determined. The results showed an average tumor weight increase of 61% in the treatment group compared with the control group, indicating a considerable trend toward significance (*p* = 0.08; Fig. 3). This result is in agreement with those of the higher CD4^+^CD25^+^/CD4^+^ ratio in the treatment group.

## DISCUSSION

Tumor microenvironment is full of immune-suppressive cytokine networks^[^^19^^,^^20^^]^. TGF-β produced in the tumor milieu either by the tumor cells or the myeloid cells^[1]^ can cause the proliferation of CD4^+^CD25^+^ Treg cells^[1]^ or the conversion of CD4^+^CD25^-^ into CD4^+^CD25^+^ Treg cells^[12,13]^, a reason that in human and murine tumors, increase of CD4^+^CD25^+^ T cells has been associated with the reduction of CD4^+^CD25^-^ T cells^[^^21^^,^^22^^]^.

Arginase I is expressed by macrophages under a variety of stimuli like parasitic antigens, Th2 cytokines, and also the cytokine TGF-β to resolve the respective infections or help healing processes^[^^23^^,^^24^^]^. In tumors, special types of macrophages, known as MDSC with the Gr-1^+^ CD11b^+^ phenotype utilize arginase to deplete arginine from the environment and suppress T cell immune responses^[25]^. It has been proposed that arginase-expressing MDSCs are capable of mediating the induction of CD4^+^CD25^+^ Treg cells^[^^15^^]^, and this capability is arginase I dependent^[^^14^^]^. Several studies have suggested increase of CD4^+^CD25^+^ regulatory T cells among the splenocytes of tumor bearing mice and its correlation with increasing tumor burden^[^^12^^,^^21^^,^^26^^]^. In our findings, no significant change was observed in crude CD4^+^CD25^+^ populations between the treatment and control groups; however, a significant reduction of CD4^+^CD25^-^ population in the treatment group was evident, which may have accounted for the increase of CD4^+^CD25^+^/CD4^+^ ratio in this group. This finding is in agreement with that of others showing that the incidence of tumors or even increase in the volume of tumors does not have any considerable impact on crude CD4^+^CD25^+^ numbers in the spleens of the affected mice. Nonetheless, it is the CD4^+^CD25^-^ population that changes towards reduction to make a homeostatic alteration in CD4^+^CD25^+^/CD4^+^ ratio as a response to increasing tumor burden^[^^21^^,^^22^^]^. This phenomenon might be due to the conversion of CD4^+^CD25^-^ T cells into CD4^+^CD25^+^ T cells^[^^12^^,^^13^^]^ as a result of enhanced TGF-β signaling caused by the protein treatment.

Elevation of CD4^+^CD25^+^/CD4^+^ ratio in the group treated with the protein is indicative of increased Treg populations among splenocytes^[^^12^^,^^21^^]^. This phenomenon can be attributed to the heightened TGF-β signaling due to WW2/WW3 protein treatment. Based on this scenario, the elevated TGF-β signaling well overshadowed the reduced arginase I expression. It can be inferred that the reduced arginase I expression could not reverse the effects exerted by the increased TGF-β signaling, as evidenced by the fact that CD4^+^CD25^+^/CD4^+^ ratio in the treatment group were not reduced or stabilized as compared with the control group.

In conclusion, application of peptides with the capacity to interact with downstream signaling cascade apparatus components is probably bound to alter the signaling cascade and bring about consequential changes in physiological parameters, which are subject to such signaling cascades. This observation holds great promise in changing the course of diseases with underlying signaling aberrations.
